# The structure and function of mitofusin 2 and its role in cardiovascular disease through mediating mitochondria-associated endoplasmic reticulum membranes

**DOI:** 10.3389/fcvm.2025.1535401

**Published:** 2025-05-30

**Authors:** Yuhu Lv, Liyuan Chen, Long Li, Ziyuan Liao, Zuoming Fang, Lin Cheng, Fenglin Peng

**Affiliations:** ^1^ College of Physical Education, Guangdong University of Education, Guangzhou, China; ^2^ Research Center for Adolescent Sports and Health Promotion of Guangdong Province, Guangzhou, China; ^3^ College of Physical Education, Xichang University, Xichang, China; ^4^ College of Physical Education and Health, Guangxi Normal University, Guilin, China

**Keywords:** mitochondria-associated endoplasmic reticulum membranes, mitofusin 2, cardioprotection, cardiovascular disease, mitochondria, endoplasmic reticulum

## Abstract

Cardiovascular disease (CVD) remains a leading cause of death globally, posing a major public health challenge. Due to the complexity of CVD's etiology, understanding its pathogenesis has been a significant challenge and research focus. In recent years, the communication between organelles has gained increasing attention, with mitochondria-associated endoplasmic reticulum (ER) membranes (MAMs) emerging as a key structural component that facilitates dialogue between the mitochondria and the ER. Numerous studies have highlighted that proteins located in MAMs may play a role in the development of CVD. Among these, mitofusin 2 (MFN2), a protein found on the outer mitochondrial and ER membranes, has garnered particular interest due to its widespread presence in MAMs. This review aims to sort out current research on MFN2, focusing on its potential involvement in myocardial protection through its mediation of MAMs. We discuss how MFN2-mediated MAMs may contribute to the protection against various CVDs, including myocardial ischemia/reperfusion injury, diabetic cardiomyopathy, dilated cardiomyopathy, pathological myocardial hypertrophy, cardiotoxicity, and heart failure. However, given the functional diversity of MFN2, the current body of research remains controversial, and further studies are urgently needed to clarify its precise mechanisms of action.

## 1 Introduction

Cardiovascular disease (CVD) is the leading cause of death from non-communicable diseases globally and represents a common endpoint for many chronic diseases (1). Effective programs for the prevention and treatment of CVD remain insufficient worldwide, primarily due to the diverse and multifactorial causes of the disease (2). The endoplasmic reticulum (ER) and mitochondria are two critical organelles involved in cellular protein production and energy metabolism, as well as in biological processes such as signal transduction, redox balance, calcium homeostasis, and apoptosis (3). It has been shown that some mitochondria and ER can be connected by tethering proteins to form mitochondria-associated ER membranes (MAMs) (4–6). MAMs are phospholipid bilayer structures composed of ER and mitochondria, including the outer mitochondrial membrane (OMM), the endoplasmic reticulum membrane, and the portion between them, and studies have shown that the distance of MAMs composed of smooth endoplasmic reticulum to mitochondria is 10–50 nm, and the distance of MAMs composed of rough endoplasmic reticulum to mitochondria is 50–80 nm (7). This structure is closely associated with the pathophysiology of CVD (8–10). Comparative analysis has shown that 1,347 proteins (approximately 96.56%) are highly conserved and expressed in human and mouse testis MAMs (11). Among these, some proteins are essential for the structural composition of MAMs, such as mitofusin 2/1 (MFN2/1), voltage-dependent anion channel (VDAC), inositol-1,4,5-triphosphate (IP3) receptor (IP3R), glucose-regulated protein 75 (GRP75), vesicle-associated membrane-protein-associated protein B, protein tyrosine phosphatase-interacting protein 51, B-cell receptor-associated protein 31, mitochondrial fission 1, and phosphofurin acidic cluster sorting protein 2. Other proteins serve regulatory functions, including calnexin, Sarco/ER calcium ion (Ca^2+^) ATPase, sigma-1 receptor, phosphatidylserine synthase, cyclophilin D, protein kinase B (AKT), mammalian TOR complex 2, long-chain fatty acid coenzyme A ligase 4, ER oxidoreductase-1*α*, and autophagy-related gene 14 (12). These proteins operate individually or in complexes, and our previous review highlighted the diverse functions of MAMs, which include, but are not limited to, the modulation of Ca^2+^ homeostasis, lipid homeostasis, mitochondrial dynamics, autophagy, mitophagy, apoptosis, and inflammation (3). Among the numerous proteins associated with MAMs, MFN2 stands out as the most specialized, being present not only on the OMM but also on the ER membrane (3, 13). Thus, MFN2 may play an important role in MAMs. In this review, we focus on the structure and function of MFN2 and its role in regulating MAMs to provide cardioprotection.

## 2 Structure of MFN2

**Figure 1:**
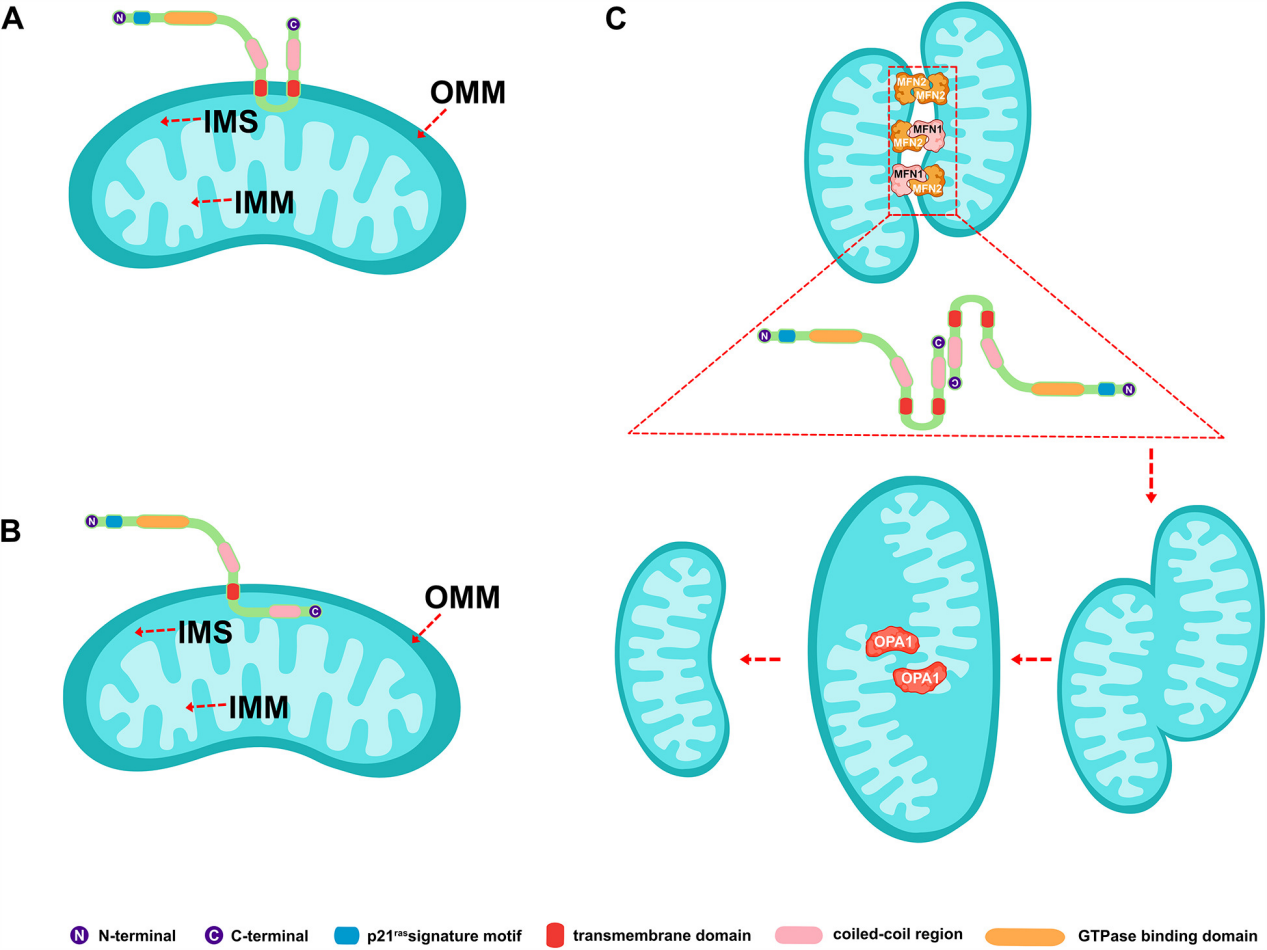
Schematic representation of the structure of mitofusin 2 and its involvement in mitochondrial fusion. **(A)** Classical MFN2 structural model. **(B)** Latest predicted MFN2 structural model. **(C)** MFN2-mediated mitochondrial fusion process. MFN2/1, mitofusin 2/1; IMM, inner mitochondrial membrane; OMM, outer mitochondrial membrane; IMS, mitochondrial intermembrane space; and OPA1, optic atrophy factor 1.

MFN2 is a highly conserved GTPase composed of 757 amino acid residues. It has both an N-terminal and a C-terminal, each exposed in the cellular matrix, playing a central role in regulating mitochondrial fusion and cellular metabolism (14–17). The protein contains an N-terminal p21ras signature motif (amino acids 77–96), a GTPase-binding domain (amino acids 93–342), two coiled-coil regions (amino acids 391–434 and 695–738), and two transmembrane domains (amino acids 605–647) that span the OMM (Figure 1A) (14, 15, 17). MFN1 is structurally similar to MFN2, except that MFN1 contains 741 amino acid residues and the GTPase-binding domain is between amino acid residues 75–336 (18, 19). A recent comparative analysis and phylogenetic reconstruction study proposed a predictive model that contradicts current models. Bioinformatics analysis revealed that fungal Fuzzy onions 1 proteins possess two predicted transmembrane structural domains, while metazoan MFN2 contains only one. This newly predicted topology of MFN2 was biochemically confirmed, with the C-terminal oxidative reduction-sensitive cysteine residue located within the mitochondrial intermembrane space (IMS). Functional experiments further supported the notion that redox-mediated disulfide bond modification within this IMS structural domain is a key regulator, driving reversible MFN2/1 oligomerization necessary for fusion (Figure 1B) (20, 21). The discovery of this structural feature provides a mechanistic basis for the coordination between MFN2/1-dependent OMM fusion and optic atrophy factor 1 (OPA1)-dependent inner mitochondrial membrane (IMM) fusion (20, 22).

## 3 Function of MFN2

It is widely accepted that sequence determines structure, which in turn determines function (23, 24). The structure of MFN2 gives it a certain function, and we combed through the functions of MFN2 reported in existing studies.

### 3.1 Involved in mitochondrial fusion

**Figure 2:**
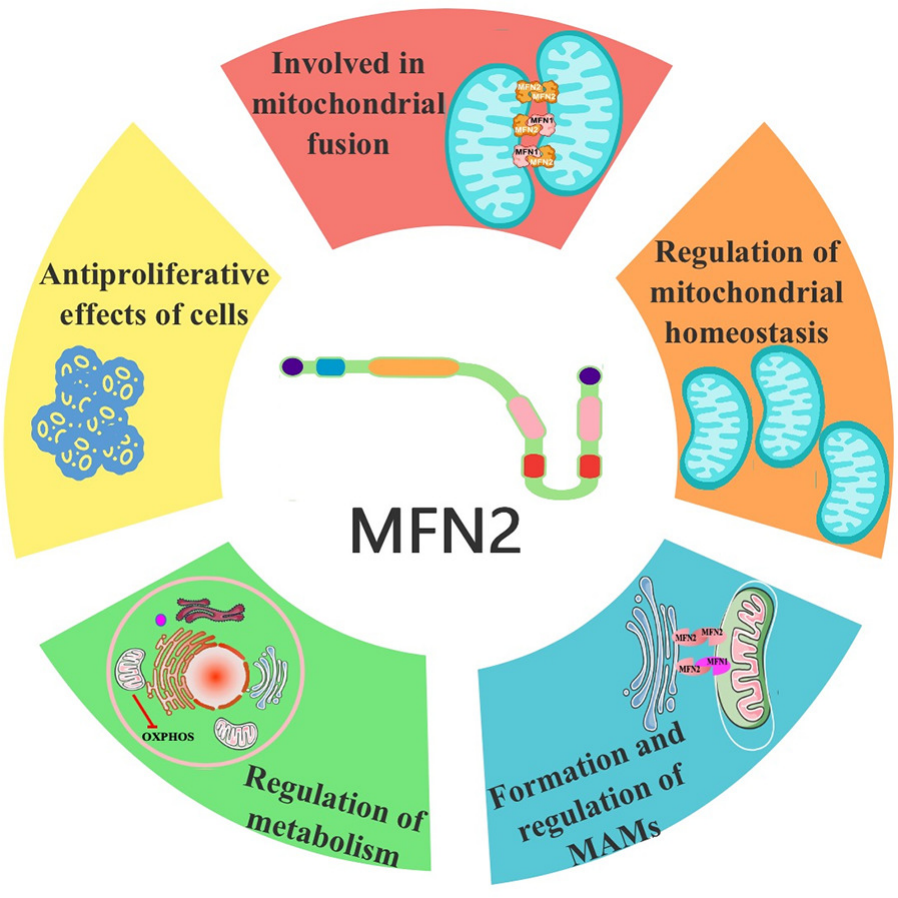
Functional schematic of MFN2. MFN2, mitofusin 2.

The mitochondrial fusion involves several proteins, including MFN2 and MFN1 on the OMM and OPA1 on the IMM. It was shown that mitochondrial phosphatase phosphoglycerate mutase 5 (PGAM5) regulates MFN2 phosphorylation, thereby protecting it from ubiquitination and degradation (25). While phosphorylation promotes mitochondrial fission and degradation, and dephosphorylation promotes mitochondrial fusion (25). MFN2 and MFN1 mediate the fusion of the OMM, while OPA1 mediates the fusion of the IMM (26, 27). The GTP hydrolyzed by these fusion proteins enables two neighboring mitochondria to fuse, allowing the sharing of mitochondrial DNA, proteins, and metabolites (27). MFN2 on one mitochondrion can form either a homodimer (i.e., MFN2 binding to another MFN2 molecule to form MFN2-MFN2 polymers) or a heterodimer (i.e., MFN2 binding to MFN1 to form MFN2-MFN1 polymers) with MFN2/1 on another mitochondrion. Studies have shown that combining MFN2 and MFN1 in a heterodimer is the most efficient mechanism for MFN2-mediated OMM fusion (28, 29). A schematic representation of mitochondrial fusion, based on the generally accepted double-transmembrane structure, is shown in Figure 1C. The mechanism of action involves the reverse parallel interaction of the coiled-coil region at the C-terminal end of MFN2 (or MFN2 on one mitochondrion and MFN1 on the other) in two mitochondria, facilitating docking. This is followed by GTP hydrolysis and, ultimately, OMM fusion (29–31) (Figure 2). The mechanism of OMM fusion based on the single-transmembrane structure of MFN2/1 remains under investigation.

### 3.2 Regulation of mitochondrial homeostasis

MFN2 also plays a crucial role in regulating mitochondrial homeostasis. A study on Charcot-Marie-Tooth type 2A revealed that mutations in the *Mfn2* gene lead to alterations in mitochondrial morphology and homeostasis (32). The activation of the MORN repeat-containing protein 4 (MORN4)-MFN2 axis regulates myocardial mitochondrial homeostasis. Mechanistically, MORN4 binds directly to MFN2 and promotes the phosphorylation of MFN2 at serine 442 via Rho-associated protein kinase 2, thereby mediating beneficial mitophagy induced by mitochondrial dynamics (33). A study in cultured neurons demonstrated that inhibition of USP30 promotes the ubiquitination of MFN2 by the E3 ubiquitin ligase (Parkin). This process separates damaged mitochondria from the healthy mitochondrial network and facilitates mitophagy, thereby removing damaged mitochondria from the cell (34). Modulation of mitochondrial morphology can likewise regulation of mitochondrial homeostasis. MFN2 also physically interacts with the protein kinase PERK. In cells ablated for *Mfn2*, sustained activation of PERK occurs under basal conditions. Silencing of PERK, however, reduces reactive oxygen species (ROS) production, normalizes mitochondrial Ca^2+^ levels, and improves mitochondrial morphology (35, 36). Similarly modulation of mitochondrial dynamics can regulation of mitochondrial homeostasis. In a vitro study in cardiomyocytes confirmed that overexpression of OPA1 and MFN2 inhibited hypoxia-induced mitochondrial division in H9c2 cardiomyocytes and reduced ROS production, thereby delaying the process of cardiomyocyte hypertrophy (37). A study in alveolar epithelial cells (A549) demonstrated that upregulation of MFN2 and OPA1 attenuates oxidative stress, mitochondrial damage, dysfunction, and mitophagy (38). Paeonol (chemically 2'-hydroxy-4'-methoxymethylphenol, a natural phenolic antioxidant extracted from the root bark of Paeonia lactiflora) stimulates mitochondrial fusion by activating the PKCε-Stat3-MFN2 pathway, protecting the heart from doxorubicin-induced injury (39). In contrast, resveratrol has been shown to restore mitochondrial quality control in myocardial ischemia/reperfusion injury via the Sirt1/Sirt3-MFN2-Parkin-PGC-1α pathway (40).

Mitophagy, the process of removing senescent or damaged mitochondria, is a key mechanism for maintaining mitochondrial homeostasis. Increased expression of MFN2 may upregulate mitophagy marker proteins, such as BNIP3l and Beclin1 (41). Both *in vivo* and *in vitro* studies have shown that activation of the MFN2/Pink1/Parkin mitophagy pathway reduces mitochondrial fragmentation in podocytes. Upregulation of MFN2 reduces puromycin aminonucleoside-induced podocyte injury, while downregulation of MFN2 limits the renal protective effects by modulating mitophagy (42). A study of atherosclerotic endothelial cells found that MFN2/Pink1/Parkin-mediated mitochondrial autophagy is critical for alleviating atherosclerosis (43). Metformin has been shown to cooperate with PINK1/MFN2 overexpression to inhibit ROS generation, reduce mitochondrial dysfunction, increase ATP generation, enhance mitochondrial membrane potential, and ultimately improve mitochondrial function, thus preventing cardiac injury (44). Research in astrocytes revealed that upregulation of MFN2 expression activates mitophagy through the PI3K/Akt/mTOR pathway, leading to the removal of damaged mitochondria in astrocytes (45) (Figure 2).

### 3.3 Formation and regulation of MAMs

*In vitro* assays, along with genetic and biochemical evidence, support the concept that MFN2 on the ER links the ER and mitochondria by forming homotypic and heterotypic complexes with MFN2 or MFN1 on the mitochondrial surface, effectively bridging the gap between the two organelles. Specifically, MFN2 on the ER interacts with MFN2 or MFN1 on the mitochondria to form either homotypic (MFN2-MFN2) or heterotypic (MFN2-MFN1) complexes, thereby linking the ER and mitochondria (20, 46). In contrast, the ablation or silencing of *Mfn2* in mouse embryonic fibroblasts and HeLa cells disrupts ER morphology, weakens the interaction between the ER and mitochondria, and reduces the efficiency of mitochondrial Ca^2+^ uptake upon stimulation by IP3 production (17). Similarly, silencing *Mfn2* in young mice reduces cardiomyocyte ER-mitochondrial contact (approximately 15 nm) by 30% and inhibits mitochondrial Ca^2+^ uptake from the neighboring ER (47). A recent study demonstrated that splicing of MFN2 generates two ER-specific variants, ERMIT2 and ERMIN2. ERMIN2 regulates ER morphology, while ERMIT2 localizes to the ER-mitochondrial interface, interacting with mitochondrial mitofusins to tether the ER and mitochondria. This interaction promotes mitochondrial Ca^2+^ uptake and phospholipid translocation. Notably, the expression of ERMIT2 ameliorated ER stress, inflammation, and fibrosis in liver-specific *Mfn2* knockout (KO) mice (13). Furthermore, a separate study showed that the mitochondrial ubiquitin ligase MITOL regulates MAMs by activating K192 ubiquitination of the MFN2 GTPase structural domain (48). MITOL also prevents ER stress-induced apoptosis through IRE1α ubiquitination on MAMs (49). MAMs are disrupted during mitophagy and reduced ER-mitochondrial contact accelerates mitochondrial degradation. This process is driven by a rapid burst of Parkin/PINK1-catalyzed MFN2 phosphorylation, which triggers p97-dependent disassembly of the MFN2 complex from the OMM, thereby separating the mitochondria from the ER (50). Additionally, MAMs can contribute to MAMs for the formation of autophagic vesicles. A reduction in MFN2 significantly disrupts MAMs and attenuates autophagy, possibly through regulation by the AMP-activated protein kinase (AMPK)-MFN2 axis (51, 52). Knockdown of endogenous *Mfn2* via stable small hairpin RNA (shRNA) expression targeting the 3′ untranslated region of *Mfn2* increases the distance between the ER and the OMM. In contrast, tyrosine phosphorylation of MFN2 regulates the distance between the OMM and ER via c-Src, leading to elevated mitochondrial Ca^2+^ levels and oxidative stress (53).

However, conflicting reports also exist. Studies in HeLa cells using shRNA to silence *Mfn2* indicate that such silencing increases the ER-mitochondrial linkage (54, 55). Additionally, the total number of ER-mitochondrial associations detected using a novel bifluorescence complementation method that labels a subset of ER-mitochondrial associations in fixed and living cells increased as MFN2 levels decreased (56). After the ablation or knockdown of *Mfn2*, there was a net increase in the number of narrow (∼10 nm) ER-mitochondrial contacts, while the number of wider (∼50 nm) contacts decreased (55, 56) (Figure 2).

### 3.4 Regulation of metabolism

An earlier study demonstrated that MFN2 is associated with mitochondrial energy metabolism. Specifically, loss of MFN2 function inhibits the oxidation of pyruvate, glucose, and fatty acids while decreasing mitochondrial membrane potential. Conversely, the gain of MFN2 function increases glucose oxidation and mitochondrial membrane potential. Mechanistically, in the *Mfn2* cause Charcot-Marie-Tooth neuropathy type 2A, loss of MFN2 function inhibits the nuclear-encoded subunits of the oxidative phosphorylation (OXPHOS) complexes I, II, III, and V, whereas MFN2 overexpression induces the subunits of complexes I, IV, and V (57). Studies on liver-specific *Mfn2* KO mice show that MFN2 connects mitochondrial and ER function with insulin signaling and is essential for normal glucose homeostasis (58). Furthermore, MFN2 influences the tricarboxylic acid cycle through sirtuin 3/isocitrate dehydrogenase 2 (NADP+) and mitochondrial activation, modulating glucose-stimulated insulin secretion in β-cells (59). In addition, MFN2 in pro-opiomelanocortin neurons regulates whole-body energy homeostasis by fine-tuning the function of the mitochondria-ER axis. In the brain, pro-opiomelanocortin neuron-specific deletion of *Mfn2* results in binge eating and obesity, accompanied by reduced energy expenditure (60). A study showed that MFN2, highly expressed in brown adipose tissue, mediates mitochondria-lipid droplet interactions, influencing lipolytic processes and overall energy homeostasis. *Mfn2* KO mice exhibit severe BAT dysfunction, impaired respiratory capacity, and a sluggish response to adrenergic stimuli (61). In contrast, both loss and gain of function of GRP75 or MFN2 significantly alter cholesterol metabolism, promoting triglyceride accumulation in hepatocytes (62). Furthermore, analysis of muscle *Mfn2*-KO mice revealed that aging-induced reductions in MFN2 underlie age-related changes in metabolic homeostasis and muscle loss (63) (Figure 2).

### 3.5 Antiproliferative effects of cells

MFN2 may also exert antiproliferative effects. Hypoxia-induced downregulation of MFN2 has been identified as a key determinant of smooth muscle cell (SMC) proliferation in pulmonary arteries (64). The mechanism may involve MFN2 counteracting cell proliferation by attenuating glycolysis. A study in cancer cells showed that MFN2 can interact with the M2 isoform of pyruvate kinase, one of the rate-limiting enzymes of glycolysis, to promote mitochondrial fusion, enhance OXPHOS, and reduce glycolysis (65). In vascular SMCs, mutations in the protein kinase A phosphorylation site on MFN2 inhibited its antiproliferative activity, an effect independent of its regulation of mitochondrial morphology (66). In cultured human vascular SMCs, GATA2 can bind to the *Mfn2* promoter region and promote MFN2 expression. Interference with GATA2 reduces MFN2 expression and impedes the proliferation of human vascular SMCs (67). Further studies showed that MFN2 inhibits glycolysis in vascular SMCs by degrading phosphofructokinase-1 and prevents neointimal hyperplasia in vein grafts (68). Another study indicated that activation-induced degradation of MFN2 is a prerequisite for T-cell entry into the cell cycle. This supports the idea that MFN2 inhibits cell proliferation, with the PI3K-AKT-mTOR pathway playing an important role in the activation-induced downregulation of MFN2 and subsequent T-cell proliferation (15). In contrast, overexpression of MFN2 effectively attenuates astrocyte proliferation and halts the cell cycle. This is accompanied by downregulation of labeling and inhibition of wound healing, as MFN2 overexpression inhibits reactive astrocyte formation by blocking the Raf1-ERK1/2 and PI3K-AKT signaling pathways (69) (Figure 2).

## 4 The role of MFN2-mediated MAMs in CVD

CVD has become the leading cause of death worldwide (70). Increasing evidence suggests that MAMs may be involved in the pathogenesis of CVD. As an essential tethering protein at MAMs, MFN2 plays a pivotal role in regulating MAM function and, consequently, influencing the disease processes associated with CVD. Furthermore, MFN2-associated strategies to mitigate CVD are being increasingly explored and reported (2, 3, 10, 71–78). In the following, we focused on the existing reports that MFN2 acts in CVD by regulating MAMs, with the aim of providing research ideas for further studies.

### 4.1 Myocardial ischemia/reperfusion (I/R) injury (MIRI)

**Figure 3:**
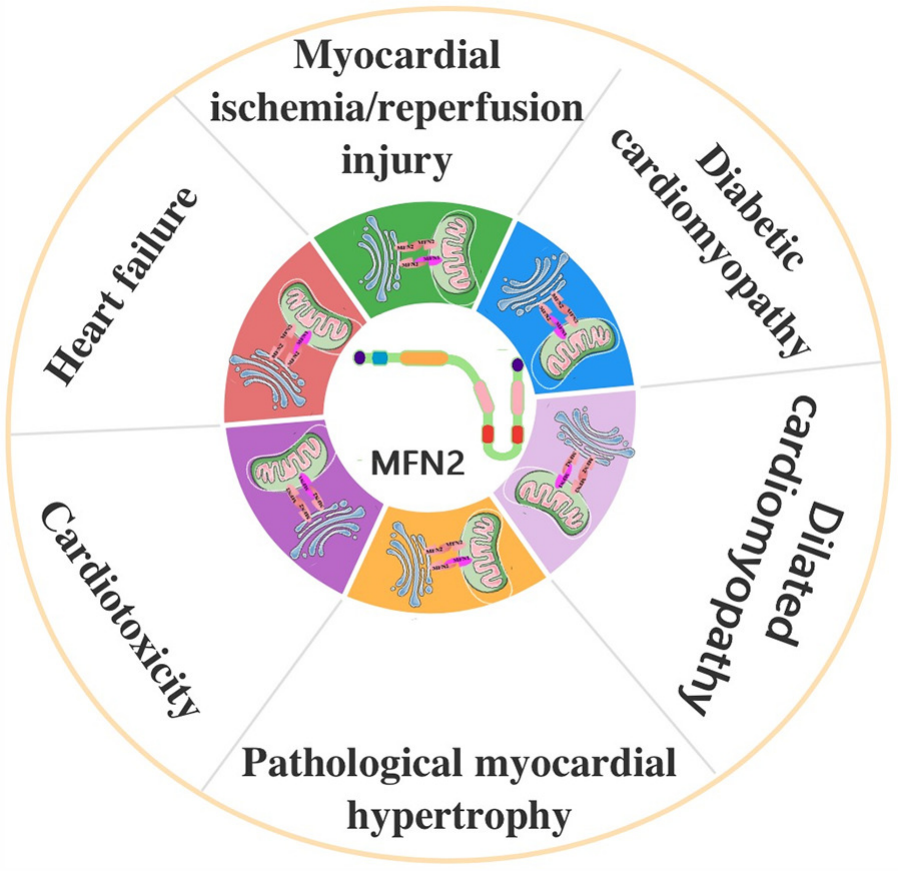
The role of MFN2-mediated MAMs in cardiovascular disease. MFN2, mitofusin 2; MAMs, mitochondria-associated endoplasmic reticulum (ER) membranes.

MIRI is widely recognized as a major contributor to cardiac dysfunction and cardiomyocyte death in coronary artery disease (3, 36). A study revealed that MFN2 acts as a key tethering junction at MAMs. In hearts from *Mfn2* and *Mfn1* KO mice, the loss of MFN2 reduces mitochondrial-ER interactions, attenuates mitochondrial Ca^2+^ overload, and protects against acute I/R injury by reducing ROS production (79). In addition, *Mfn2*-KO adult cardiomyocytes were found to be protected against various stimuli that induce cell death, and *Mfn2* KO hearts exhibited better recovery following reperfusion injury (80). Similarly, research using a mouse model of MIRI showed that downregulation of MFN2 expression promotes cardiomyocyte proliferation and inhibits apoptosis (81). However, other studies present contrasting findings. In a different application, ischemic preconditioning significantly increased MFN2 expression in skeletal muscle during total knee arthroplasty combined with tourniquet use. This increase partially protected postoperative quadriceps strength by enhancing mitochondrial fusion proteins and preventing tourniquet-induced I/R injury, suggesting that MFN2 upregulation may have protective effects against I/R injury (82). Additionally, the upregulation of MFN2 expression through MiR-93 demonstrated inhibition of myocardial apoptosis in rats with MIRI (83). In another study, the knockdown of *Mfn2* using shRNA prevented the fusion of autophagosomes with lysosomes in neonatal cardiomyocytes. Re-expression of *Mfn2* restored this fusion, whereas cardiac-specific *Mfn2* KO mice, which exhibit abnormal cardiac mitochondria and cellular metabolism, were more susceptible to I/R challenges (84) (Figure 3). This may be related to the course of the disease, as evidenced by the fact that these effects may play an isotropic or diametrically opposed role in different courses of the same disease (85, 86). For example, it has been shown that increasing autophagy during ischemia relieves MIRI and increasing autophagy during reperfusion increases MIRI (87–89). It may also be related to the multiple roles of MFN2, as evidenced by differences in the degree of each role, which ultimately presents a combination of roles, i.e., a balanced result of the various roles. It was suggested, for instance, that physiologic autophagy may play a role in protecting the myocardium from MIRI, whereas excessive autophagy exacerbates MIRI (90–93). The above studies present conflicting results, highlighting ongoing controversy in this area. These discrepancies underscore the need for further research to elucidate the underlying mechanisms, especially in CVD that are comorbid with other diseases. For example, upregulation of AMPK/MFN2-dependent mitochondrial fusion has been shown to reduce MIRI in diabetic hearts (94).

### 4.2 Diabetic cardiomyopathy (DCM)

DCM is a distinct subtype of CVD observed in patients with type 1 or type 2 diabetes mellitus. It is characterized by alterations in myocardial structure and function (36). In cardiomyocytes from mice with DCM, increased connectivity between the sarcoplasmic reticulum and mitochondria has been observed, representing an enhanced formation of MAMs (95). A study involving obese diabetic (db/db) mice and lean control (db/+) mice showed excessive mitochondrial fragmentation and significantly reduced MFN2 expression in the hearts of 12-week-old diabetic (db/db) mice (96). Notably, reconstitution of *Mfn2* in diabetic hearts inhibited mitochondrial fission and prevented the development of DCM (96). Further research has demonstrated that MFN2 is crucial in high glucose-induced ER stress (ERS) in atrial cardiomyocytes. High glucose levels upregulate ERS, mitochondrial oxidative stress, and the MAMs-enriched proteins GRP75 and MFN2 in atrial myocytes *vitro*. Silencing *Mfn2* prevents mitochondrial dysfunction caused by mitochondrial Ca^2+^ overload, thereby reducing ERS-induced cardiomyocyte death (97). In HL-1 cells under ERS conditions, silencing *Mfn2* reduced Ca^2+^ transfer from the ER to mitochondria, and electron microscopy confirmed that *Mfn2* siRNA significantly disrupted ER-mitochondrial tethering in ER-injured HL-1 cells (97). Additionally, Paeonol was found to stimulate mitochondrial fusion through the PKCε-Stat3-Mfn2 pathway, protecting the heart from doxorubicin-induced injury (39). The pathogenesis of DCM is also believed to be linked to AMPK inactivation. Under energy stress, AMPK activation triggers mitochondrial fission and autophagy, increasing the number of MAMs. During mitochondrial fission, a large proportion of AMPK translocates from the cytoplasm to the MAMs, where it interacts with MFN2. This interaction initiates mitochondrial autophagy, contributing to cardiac protection (36, 52). In contrast, although metformin is widely recommended as a first-line treatment for type 2 diabetes, and its ability to reduce the risk of CVD in these patients remains under investigation, evidence suggests that it may reduce the incidence of myocardial infarction in patients with diabetes. This effect is thought to be mediated through AMPK activation (98) (Figure 3). Since patients with CVD may have other diseases in combination, related comprehensive studies can be added to future research to clarify the prevention and treatment of related diseases.

### 4.3 Dilated cardiomyopathy

Dilated cardiomyopathy is a CVD in which the walls of one of the heart's chambers become dilated and thin, resulting in the heart's inability to pump blood properly. MFN2 has been implicated in various CVDs, including I/R injury, heart failure, and dilated cardiomyopathy (99, 100). Genetic analyses have linked MFN2 dysfunction to dilated cardiomyopathy (101). Mitochondrial fusion is essential for maintaining normal mitochondrial morphology and the proper respiratory and contractile function of the heart. Conditional ablation of *Mfn1* and *Mfn2* in the adult heart induces mitochondrial fragmentation, cardiomyocyte dysfunction, and impaired mitochondrial respiration, ultimately leading to rapidly progressive and lethal dilated cardiomyopathy (102). Further studies have shown that the accumulation of morphologically and functionally abnormal mitochondria induces respiratory dysfunction, which leads to dilated cardiomyopathy in *Mfn2*-KO mouse embryonic fibroblasts and cardiomyocytes, as well as in Parkin-KO *Drosophila* heart tubes (103) (Figure 3). While these findings indicate a clear association between MFN2 and dilated cardiomyopathy, further research is needed to elucidate the underlying mechanisms fully.

### 4.4 Pathological myocardial hypertrophy

MAMs may play a critical role in pathological myocardial hypertrophy. A significant reduction in ER-mitochondrial contact has been observed in norepinephrine-induced cardiac hypertrophic cells. This reduction leads to decreased Ca^2+^ uptake, AKT activation, glucose uptake, and mitochondrial oxygen consumption in response to insulin (104). Earlier studies have shown that *Mfn2* expression is downregulated in hypertrophied hearts, with its expression level being linked to both the etiology and time course of hypertrophy (105). Consistent with this, *Mfn2*-KO mice exhibited moderate cardiac hypertrophy and mild functional deterioration, suggesting mild mitochondrial dysfunction (80). Furthermore, upregulation of MFN2 has been shown to inhibit angiotensin II-induced cardiac hypertrophy, with the inhibition of AKT activation playing a significant role in this process. These findings suggest that MFN2 is a key protein regulating cardiomyocyte hypertrophy (106). Additionally, hypertrophied myocardium exhibits a distinct microRNA (miRNA) expression profile compared to normal myocardium, with miRNA-20 promoting cardiomyocyte hypertrophy by reducing *Mfn2* expression (107). In contrast, upregulation of *Mfn2* expression ameliorated cardiac hypertrophy induced by angiotensin II (107). The luciferase reporter system has confirmed that *Mfn2* is a target gene of miR-195-5p, which negatively regulates *Mfn2* expression in H9c2 cells and promotes cardiac hypertrophy (108). Mechanistically, miR-17-5p may also contribute to cardiac hypertrophy by inhibiting *Mfn2* expression, activating the PI3K/AKT/mTOR pathway, and inhibiting autophagy (109). Analogously, an opposing study indicated that lncRNA ZNF593-AS inhibits cardiac hypertrophy and myocardial remodeling by upregulating MFN2 expression (110). Similarly, ubiquitin-specific peptidase 2 overexpression mediates deubiquitination to upregulate MFN2, attenuating Ca^2+^ overload-induced mitochondrial dysfunction and cardiac hypertrophy (111). In contrast, 8 weeks of aerobic exercise and choline intervention inhibited myocardial MFN2 expression, attenuated cardiac hypertrophy, and improved cardiac function in damaged cardiac tissue after aortic constriction (112). However, it has also been shown that moderate exercise cannot cause changes in MFN2 expression in adult spontaneously hypertensive rats (113). Additionally, transient receptor potential vanilloid 1 (TRPV1), a nonselective cation channel, may also be involved in pathological cardiac hypertrophy. One study suggested that TRPV1 activation exacerbates hypoxia/reoxygenation-induced apoptosis in H9c2 cells via Ca^2+^ overload and mitochondrial dysfunction (114). In contrast, in a phenylephrine-treated model of cardiomyocyte hypertrophy, TRPV1 activation reduced mitochondrial ROS, decreased cardiomyocyte size, and improved mitochondrial function by promoting the formation of MAMs. Notably, interfering with *Mfn2* via single-stranded RNA interference or silencing *Mfn2* blocks the function of TRPV1 (115). Similarly, disrupting MAM formation via si*Mfn2* abrogates the protective effects mediated by TRPV1 (115) (Figure 3). These findings suggest that MFN2 may function through its regulated MAMs. Because of the multiple roles of MFN2, further studies are needed to elucidate its specific mechanisms and to determine whether MFN2 acts through the regulation of MAMs or whether the disruption of the structure of MAMs restricts TRPV1 from acting accordingly. This will help to clarify the therapeutic targets for this disease.

### 4.5 Cardiotoxicity

Sorafenib, an antitumor agent, induces cardiotoxicity, resulting in cardiomyocyte necrosis. The mechanistic basis of this effect may involve sorafenib-induced inactivation of mTOR and the activation of transcription factor EB, which translocates to the nucleus and promotes mitophagy. This process leads to the degradation of MFN2. Further studies have shown that both global and cardiac-specific overexpression of MFN2 can suppress cardiac dysfunction and inhibit cardiomyocyte necrosis. This protective effect occurs through inhibiting the MAM-calmodulin-dependent protein kinase II delta-RIP3/mixed-lineage kinase domain-like protein pathway, which is implicated in sorafenib-induced cardiomyocyte necrosis (116). Targeting MFN2-mediated mitochondrial fusion may offer dual therapeutic benefits in doxorubicin-based chemotherapy by protecting against cardiotoxicity and enhancing its antitumor efficacy through metabolic shifts (117). A similar study revealed that total flavonoids of Selaginella tamariscina (P.Beauv.) Spring (a perennial herb that belongs to the genus Selaginella in the family Selaginellaceae, contains abundant flavonoid bioactive substances) ameliorated doxorubicin-induced cardiotoxicity by attenuating mitochondrial dysfunction and ERS by activating the MFN2/PERK pathway (118). Additionally, MFN2 may play a role in heavy metal-induced cardiotoxicity by modulating MAMs (4). A study involving sheep hearts revealed that exposure to heavy metals such as molybdenum and/or cadmium led to myocardial morphological damage, impaired oxidative function, and a significant decrease in mitochondrial Ca^2+^ levels. An increase in the distance between MAMs and disruption of the MAM structure accompanied this. Furthermore, lower MAM-related genes (including IP3R, FUNDC1, MFN2, and VDAC1) were observed. The findings suggest that molybdenum and/or cadmium induce cardiotoxicity via the MFN2 pathway, leading to the disorganization of MAMs (119). In contrast, the sirtuin1 agonist resveratrol inhibits cardiomyocyte apoptosis by upregulating MFN2, antagonizing oxidative stress, and alleviating doxorubicin-induced cardiotoxicity (120). Similarly, trophoblast stem cell-derived exosomes have been shown to attenuate doxorubicin-induced cardiotoxicity through anti-apoptotic effects and improve mitochondrial fusion by increasing MFN2 expression (121) (Figure 3). Therefore, *Mfn2* may be a key target for the treatment of cardiotoxicity, and methods such as promoting the expression of *Mfn2* or targeting the administration of certain Mfn2 protein preparations may be developed in the future to alleviate cardiotoxicity. Thus, *Mfn2* is a key target for the treatment of cardiotoxicity, which can be alleviated by promoting MFN2 expression.

### 4.6 Heart failure

Heart failure has become an increasingly significant public health issue due to the aging global population. Despite substantial progress in understanding the disease, the development of effective therapies for heart failure continues to face several challenges (122). MAMs may provide viable targets for intervention in heart failure treatment (6). Dysfunction of MFN2, a key protein involved in forming and regulating MAMs, has long been associated with CVDs, including heart failure (99, 100). In the failing heart, an accumulation of MFN2 and MFN1 typically occurs in response to proteasome inactivation. Compared to sham-treated animals, cardiac Mfn1, Mfn2, and Dnm1l mRNA levels were significantly lower (123). This suggests that the elevated levels of MFN1 and MFN2 observed in the failing heart may result from impaired proteasome activity, consistent with findings from animal models of heart failure and human cardiac failure (124, 125). Furthermore, Mfn1/Mfn2 double-KO mice develop heart failure during embryonic life, leading to death (102) (Figure 3). While these findings highlight the role of MFN2 in heart failure, relevant reports remain scarce, indicating the need for further research to uncover the specific mechanisms involved. Future studies are especially needed to clarify whether it is the action of MFN2 or MFN1 alone or the result of the combined action of MFN2 and MFN1.

## 5 Conclusion

MFN2 is a highly conserved GTPase comprising 757 amino acid residues, widely distributed in the outer mitochondrial and ER membranes. MFN2 plays a crucial role in mitochondrial fusion, regulation of mitochondrial homeostasis, formation, modulation of MAMs, modulation of cellular metabolism, and the antiproliferative effects on cells. We have found that MFN2-mediated MAMs may play significant roles in a variety of CVDs, including MIRI, DCM, pathological myocardial hypertrophy, cardiotoxicity, and heart failure. However, due to the diverse range of functions of MFN2, its role in these diseases is not always consistent. Some studies report opposing findings, suggesting that understanding its precise role in these conditions remains unclear. This highlights the need for further research to elucidate the mechanisms by which MFN2 contributes to these diseases.
